# Statistical shape modeling of the geometric morphology of the canine femur, tibia, and patella

**DOI:** 10.3389/fvets.2024.1366827

**Published:** 2024-07-10

**Authors:** Jeremy Huart, Antonio Pozzi, Jason Bleedorn, Tung-Wu Lu, Sebastian Knell, Brian Park

**Affiliations:** ^1^Clinic for Small Animal Surgery, Department for Small Animals, Vetsuisse Faculty University of Zurich, Zürich, Switzerland; ^2^Department of Veterinary Clinical Sciences, Colorado State University, Fort Collins, CO, United States; ^3^Department of Biomedical Engineering, National Taiwan University, Taipei, Taiwan

**Keywords:** statistical shape model, SSM, morphology, canine, femur, tibia

## Abstract

Bone morphometry varies among dogs of different sizes and breeds. Studying these differences may help understand the predisposition of certain breeds for specific orthopedic pathologies. This study aimed to develop a statistical shape model (SSM) of the femur, patella, and tibia of dogs without any clinical orthopeadic abnormalities to analyze and compare morphological variations based on body weight and breed. A total of 97 CT scans were collected from different facilities and divided based on breed and body weight. The 3D models of the bones were obtained and aligned to a coordinate system. The SSM was created using principal component analysis (PCA) to analyze shape variations. The study found that the first few modes of variation accounted for a significant percentage of the total variation, with size/scale being the most prominent factor. The results provide valuable insights into normal anatomical variations and can be used for future research in understanding pathological bone morphologies and developing 3D imaging algorithms in veterinary medicine.

## 1 Introduction

Dogs have specific anatomical and physiological variations among breeds, body weight, and size (1, 2), that may predispose to pathologies. For example, brachycephalic dogs are subject to respiratory difficulties partially due to the shape of their skull (3). While some research has been done in small groups of dogs (4–7), no studies have provided a large-scale dataset to investigate bone anatomical variations among breeds or body weight. Since bone morphology has been shown to influence the development of certain diseases such as medial patellar luxation (MPL), large-scale methodologies to investigate bone geometry have a high potential for the prevention and treatment of these diseases (8).

Geometric morphology is the study of shape assessment using landmarks. It looks at the shape as a whole, rather than using a restricted set of anatomical parameters *per se*. There is limited information about the geometric morphology of the femur, tibia, and patella in dogs. Palierne et al. (9) measured different anatomical parameters for the normal femur. Savio et al. (10) and Soparat et al. (11) reported similar femoral varus angle (FVA) for large breed dogs (5.5°) and Pomeranian (5.85°). Similar studies have reported data for proximal tibia, but only for a single or two specific breeds (6, 12, 13). The relationship between geometric bone morphology and different orthopedic diseases is yet to be extensively investigated.

The canine hind limbs are subject to very common diseases such as hip dysplasia (HD), cranial cruciate ligament disease (CCLD), medial patella luxation (MPL), and osteochondrosis (OC) (14–16). Some studies have shown the correlation between the shape of the bones and the prevalence of these diseases by analyzing various anatomical bone measurements (17–20). The main regions susceptible to pathological variation are the extremities of the bones such as the femoral head and condyles, tibia plateau and the small bones such as the patella due to the previously mentioned disease (14–16). However, these anatomical variations can be very specific for certain breeds. The previously mentioned studies would suggest that most of the variations are in size, thickness and curvature of the bones but the specific localization and type of geometrical variation has not been investigated.

Statistical shape model (SSM) is a computational technique used in human medicine to study geometric bone morphology, and disease pathogenesis and perform medical imaging analysis, automatic and semi-automatic segmentation, and electrocardiogram simulation among other applications (21–31). Using principal component analysis (PCA), it can provide a list of the main geometrical properties (or variation parameters) of a set of 3D bone models, also known as modes, the main ones often being the size and thickness (22). PCA allows to analyze and visualize the complete 3D models by describing their shape with a mathematical algorithm, instead of quantifying each length, angle and size manually (10–13). This method provides a more global understanding of bone geometry and does not limit our understanding to local changes (21). SSM does not provide any information on the internal morphology (i.e., cortical thickness), the bone axis or joint angles which are parameters often used by clinicians. It only describes the shape of the bone with a mathematical equation. Based on our knowledge, SSM has never been used to research bone morphology in dogs.

This study aims to develop an SSM of the femur, patella, and tibia of dogs without any clinical orthopeadic abnormalities and to analyze and compare the morphological variations based on body weight and breeds. These data will provide a better understanding of the normal anatomical variations and can be used as a baseline for future studies, investigating morphologies of pathological bones, and developing and validating SSM-based 3D image reconstruction algorithms (32, 33).

## 2 Materials and methods

### 2.1 Data sample

A total of 97 computed tomography scans (CT) were collected retrospectively from three different facilities (University of Zürich, Switzerland, and University of Wisconsin, United States, University of Florida, United States). Inclusion criteria was the presence of at least the femur, patella, and proximal part of the tibia. Exclusion criteria was the presence of any pathologic abnormalities seen in the clinic or on the CT by experts, such as osteoarthritis, HD, MPL, osteochondrosis or previous surgery. This study included CT scans of both cadaveric limbs and live dogs.

### 2.2 3D model reconstruction

All hindlimbs were scanned with 0.8-mm slice thickness with 512×512 matrix, pixel size ranging between 0.1927 mm and 0.473 mm (University of Florida—Model Toshiba, University Zürich—Brilliance CT, Philips AG, Zurich, Switzerland, and University of Wisconin-Model GE Medical Systems) The images were manually segmented using an open-source software (3D Slicer). Initial segmentation was performed using a threshold value of 350, followed by performing a smoothing using a built-in function for closing (fill holes) with kernel size of 3.0 mm. Each bone (femur, patella, tibia, and fabella) was segmented separately to create an individual model.

### 2.3 Coordinate system application and meshing

The models were meshed using the engineering software Geomagic WRAP (Geomagic, Inc., Research Triangle Park, NC) to obtain 3D models of reduced size and uniformly sampled vertices (target edge length 2 mm). Complete bones and partial models (proximal distal part of the femur and proximal part of the tibia) are created for evaluations. All femur models were cut to isolate the region of interest with uniform length. Due to large variation of length and size, the length of the partial femur model was normalized to be two times the length of the condyles (partial distal model) and femoral head (partial proximal model). The length was measured to be the diameter of a sphere fitted in the condyles and the diameter of a sphere fitted in the femoral head. The partial proximal tibia model was cut at the most distal part of the tibial tuberosity (34–37).

The models were aligned to an anatomical coordinate system based on anatomic landmarks, similar to previous joint kinematics studies (38). The origin was centered on the point equally distant to the center of the two condyles. Where the center was determined by registering a sphere to the geometry of the femoral condyle. The Y-axis was set to be parallel to the anatomical axis of the femur in a proximal-distal direction, with a positive direction going proximal. The Z-axis was set to be in a latero-medial direction, with the positive direction going medial. The X-axis was then calculated to be perpendicular to the other two axes. For each group and set of bones (left and right femur, tibia and patella, and the different groups), reference/template models were created. The template models were created from the median bone model, remeshed to have uniform vertices of 2 mm (39). All the training models were registered to the template model using the nearest neighbor algorithm (MATLAB and Statistics Toolbox Release R2020a, The MathWorks, Inc., Natick, MA.) to unify the number of faces and vertices.

### 2.4 Statistical shape model

**Figure 1:**
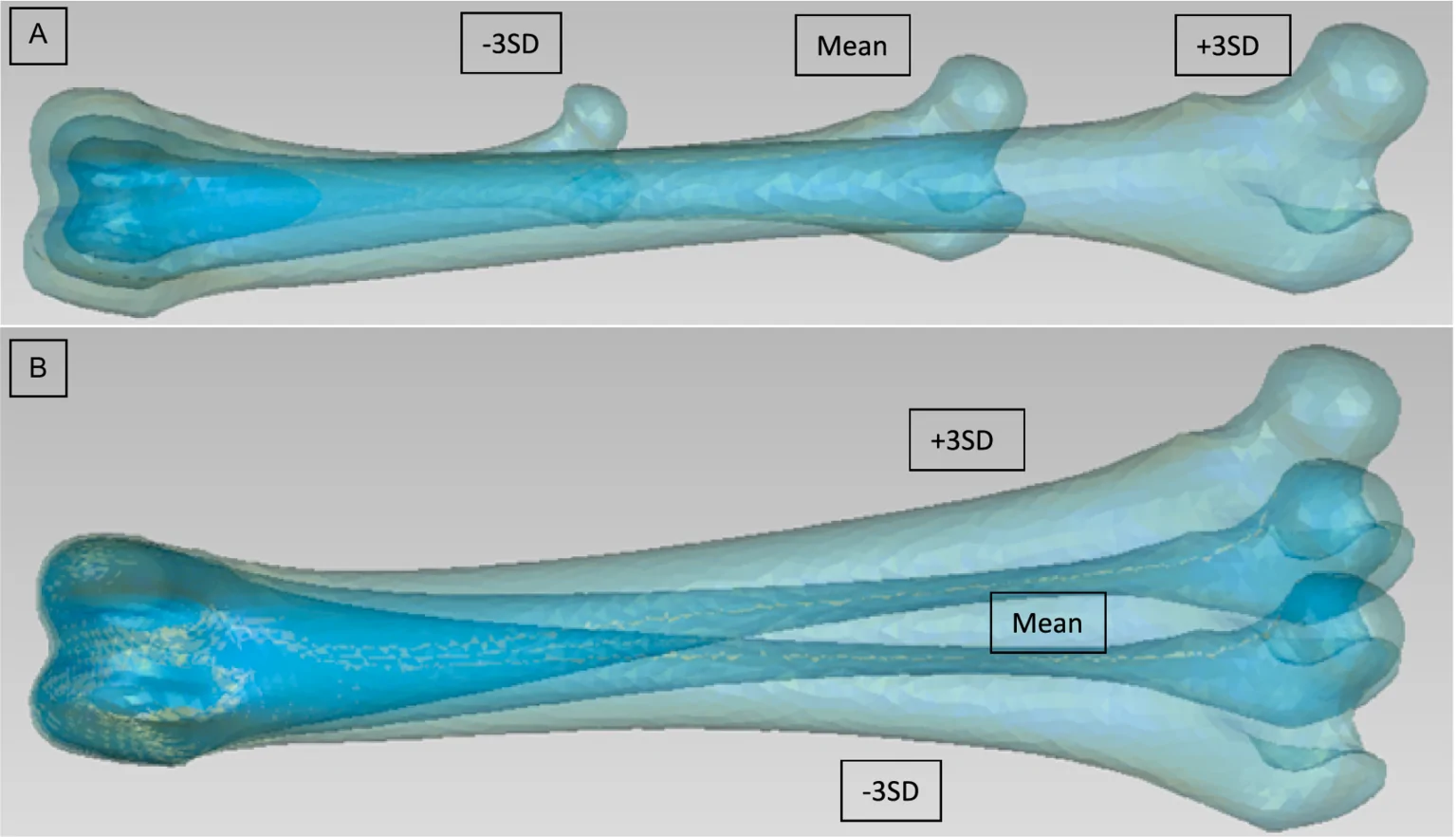
First two modes for the PCA of the left femur from Group 1 [**(A)** Mode 1 viewed from cranial, **(B)** Mode 2 viewed from cranial]. They are for each mode 3 models to be seen: Mean model and ±3 SD.

**Figure 2:**
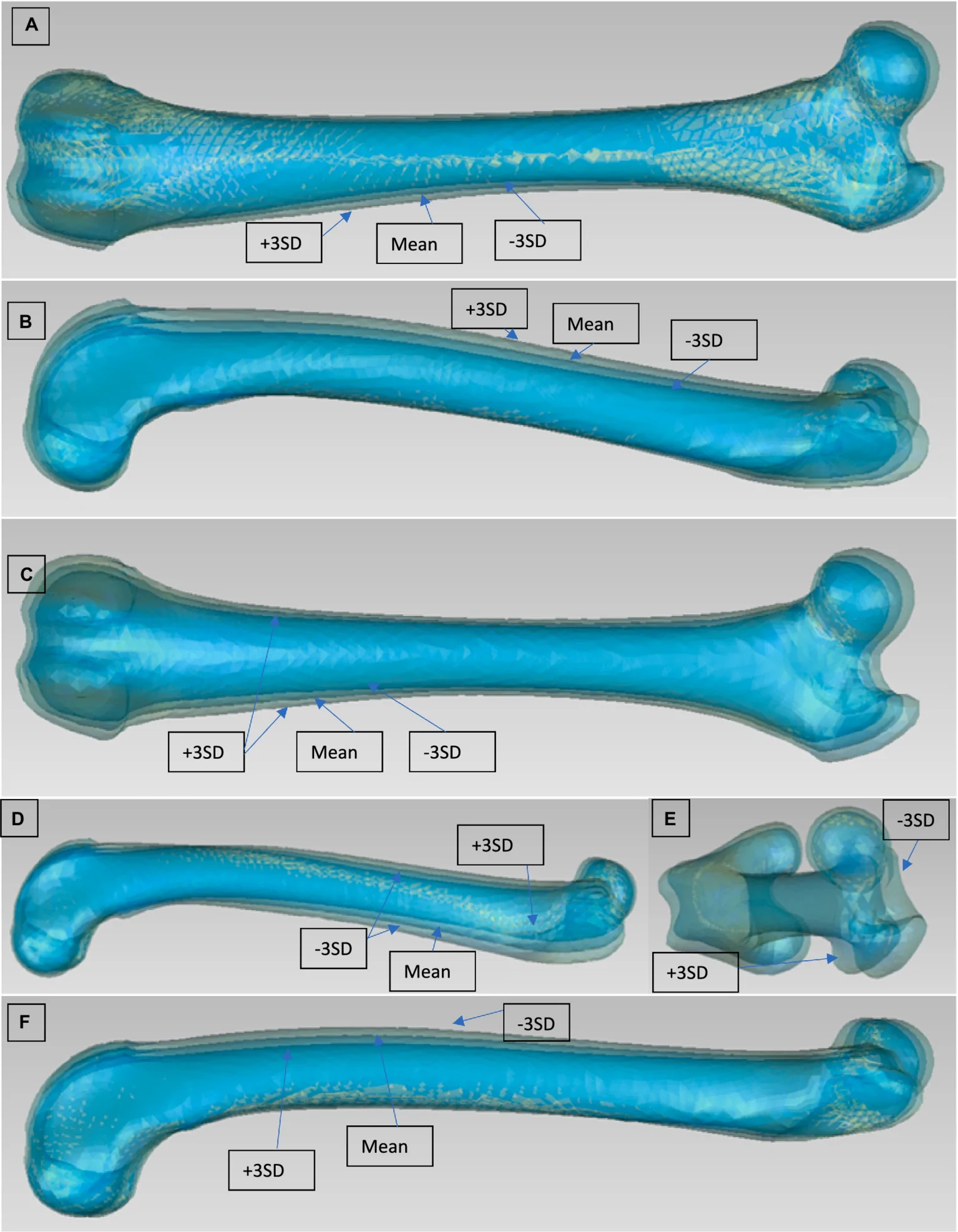
Modes 2, 3, 4, and 5 for the PCA of the left femur [**(A)** mode 2 viewed from cranial after realignment, **(B)** mode 3 viewed from lateral, **(C)** mode 3 viewed from crainal, **(D)** mode 4 viewed form lateral, **(E)** mode 5 viewed from cranioproximal, **(F)** mode 5 viewed from lateral]. They are for each modes 3 Models to be seen: Mean model and ±3 SD (only the most external model is described, then comes the mean model and the opposite model).

**Figure 3:**
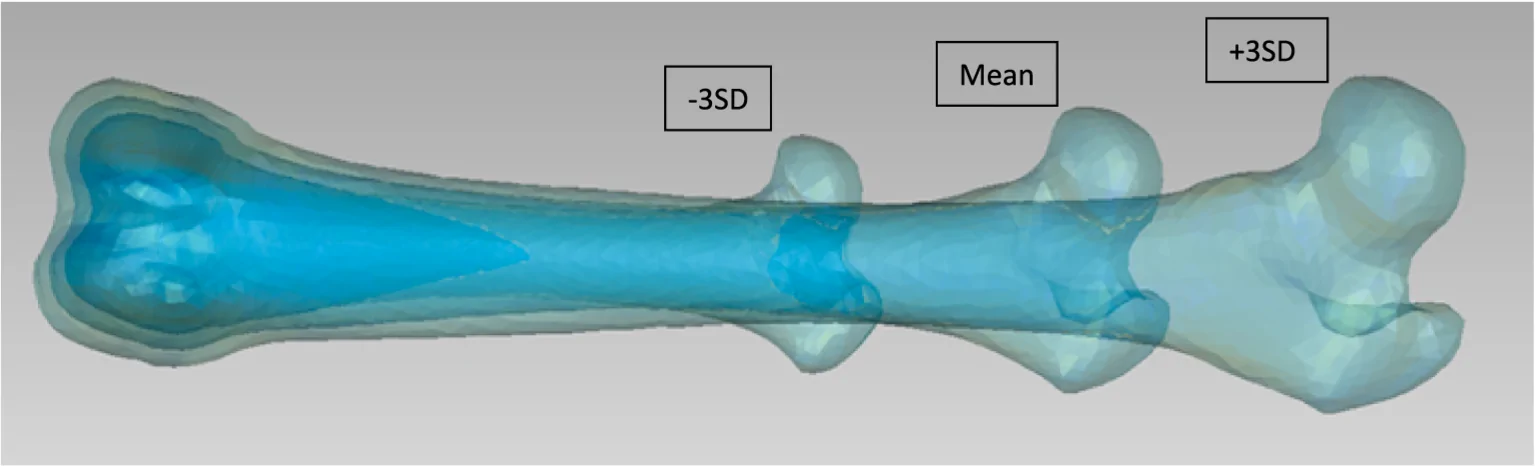
First SSM mode for the left femur from Group 2 (viewed from cranial). They are 3 Models to be seen: Mean model and ±3 SD.

**Figure 4:**
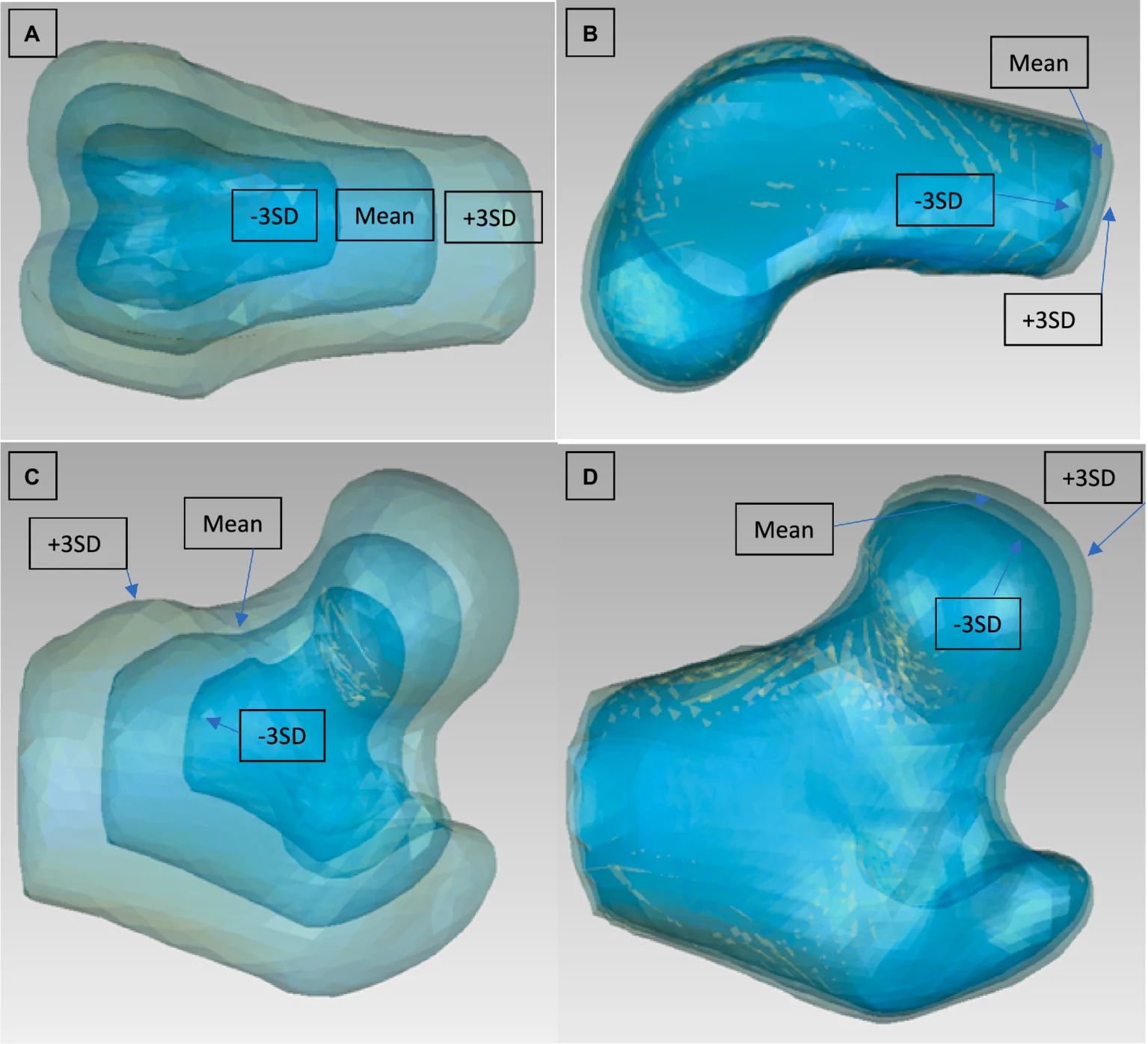
Modes 1 and 3 from the PCA of the left femur condyles [**(A)** mode 1 viewed from cranial, **(B)** mode 3 viewed from lateral] and modes 1 and 2 from the PCA of the left femoral head [**(C)** mode 1 viewed from cranial, **(D)** mode 2 viewed from cranial]. They are for each mode 3 Models to be seen: Mean model and ±3 SD.

**Figure 5:**
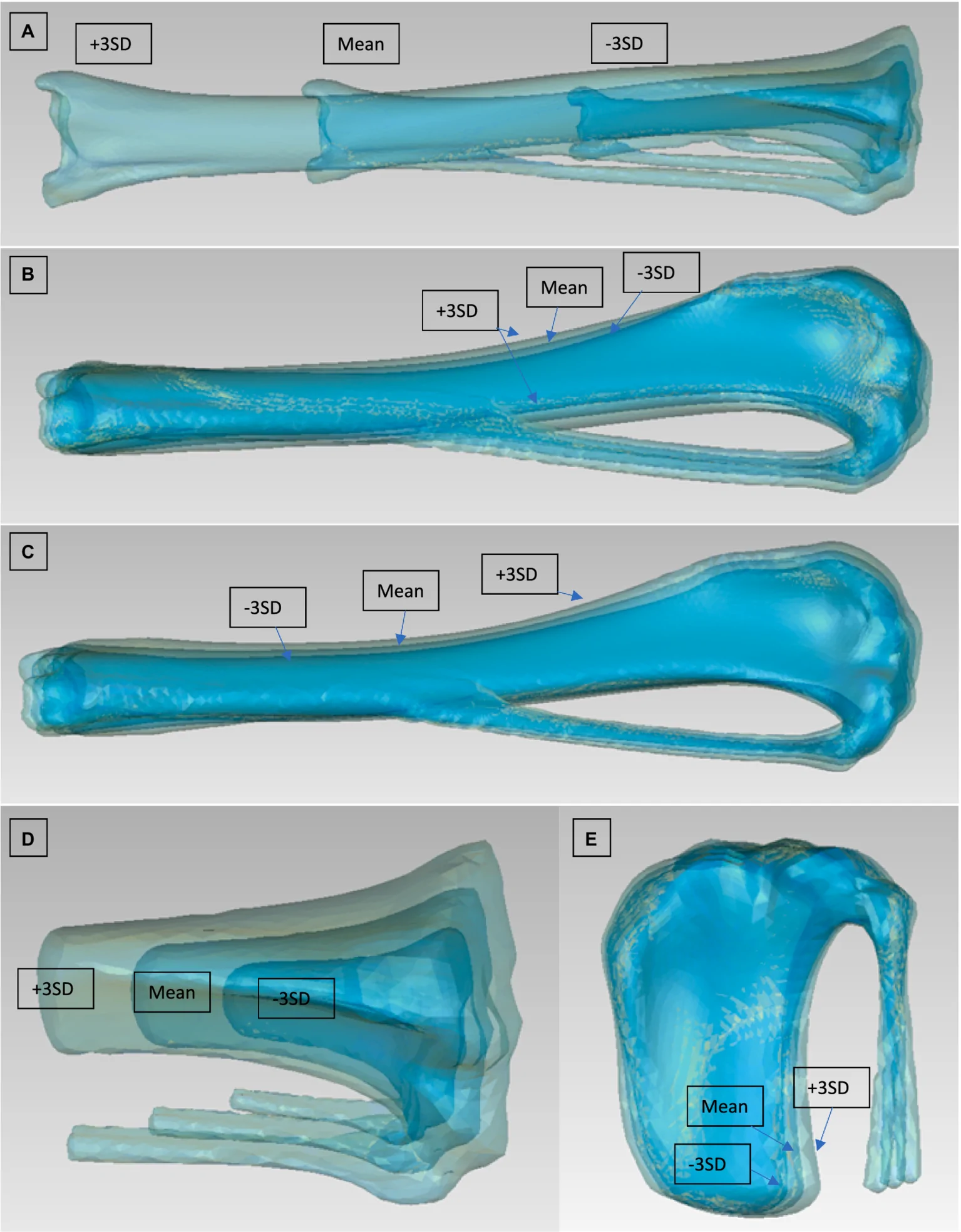
Modes 1, 4, and 5 from the PCA of the left tibia [**(A)** mode 1 viewed from cranial, **(B)** mode 4 viewed from lateral, **(C)** mode 5 viewed from lateral]. Modes 1 and 4 from the PCA of the left tibia plateau [**(D)** mode 1 viewed from cranial, **(E)** mode 4 viewed from lateral]. They are for each mode 3 Models to be seen: Mean model and ±3 SD.

**Figure 6:**
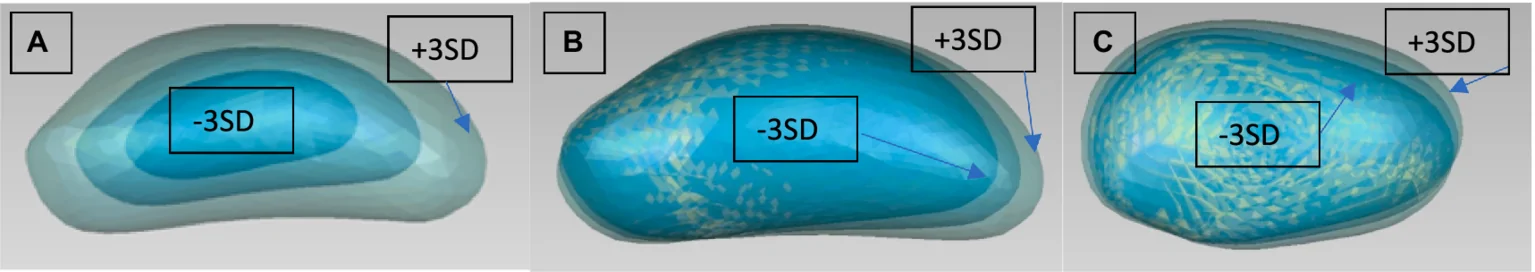
Modes 1, 2, and 3 from the PCA of the left patella [**(A)** mode 1 viewed from lateral, **(B)** mode 2 viewed from lateral, **(C)** mode 3 viewed from cranial]. They are for each mode 3 Models to be seen: Mean model and +/− 3 SD.

Once all the training models were registered and aligned to the template model, PCA was performed. This operation generated the results as the shape variation and Modes for individual bones. A Mode was defined as the modification of the mean model due to the variation of only one component of the PCA. This allowed individual analysis of each component. The Modes were numbered 1 to 5 based on the magnitude of variation that was detected in the bones. For analysis, the data for the femur were divided between body weight groups, breed, and bones (Table 1). The specific bone regions analyzed were the femoral condyle, femoral head, and the tibial plateau. A realignment of the femur model was performed with Geomagic WRAP to obtain more detailed results in some cases, such as for interpretation of the Mode 2. Observation of the models at different alignment was used to obtain more insight in localization of the deformity in the different Modes. They were aligned based on the shape of their extremities (for example: the second Mode of the complete femur). A total of 33 SSMs (mean, ±3 standard deviations) representing the first 5 Modes from the PCA were generated for visual evaluations (Figures 1–6) (25, 29).

**Table 1:** Group composition and number of samples for the different models and groups.

Groups	Composition	Number of cases	Number of breeds
Complete Femur Group 1	Whole set	61	30
Complete Femur Group 2	Weight 0–10 Kg	21	8
Complete Femur Group 3	Weight 10–25 Kg	18	17
Complete Femur Group 4	Weight > 25 Kg	22	15
Complete Femur Group 5	Chondrodystrophic Breeds	18	5
Complete Femur Group 6	Retrievers	13	2
Femur Condyles	Distal end of the femur, including the femoral condyles.	61	30
Femoral Head	Femoral head	61	30
Tibia	Complete tibia	30	13
Tibia plateau	Proximal end of the Tibia, including the Tibia plateau	72	30
Patella	Complete patella	85	30

## 3 Results

From 97 samples, 61 models without any presence of clinical orthopeadic abnormalities of complete femur with head and condyles, 30 complete tibia, 72 tibia plateaus and 85 patella were collected after exclusion of pathological conditions (*n* = 11), from 41 different type of breeds (Tables 1, 2). Groups 1 through 6 are separated by weight class and breed for complete femur. The different weight classes were set as follows: dogs from 0 to 10 kg, dogs from 10 to 25 kg and dogs greater than 25 kg body weight. The breed groups consisted of 2 groups: one for the retrievers (Golden and Labrador) and one for the chondrodystrophic breeds (French bulldog, Maltese, Dachshund, Bichon Frise, Beagles). Groups 7 and 8 were separated to evaluate partial femurs (femoral condyles and femoral head). Due to limited sample collection for complete tibia, separate evaluation for breed and weight class were not performed. Group 9 and 10 were created for complete tibia and partial tibia (proximal end of the tibia) for analysis. The last SSM group is the patella. The dog population incorporated in this study has the media age of 10 years (range 0.5–17), the median weight of 27.4 Kg (range 3–65) with 30 different with type of breeds (Table 2).

**Table 2:** Every breed and number of dogs included in the study (some dogs only had one type of bones).

Breed type	Number of cases	Breed type	Number of cases		Number of cases
Retriever	24	American Staffordshire Terrier	1	Bernese Mountain Dog	1
Mixed breed	18	Shetland sheepdog	1	mops	1
Beagle	10	Anatolian shepherd	1	ca de Bou	1
Bulldog	3	Great Dane	1	perro de agua espanol	1
German Shepherd	3	Germann Boxer	1	Chinese crested dog	1
Springer spaniel	2	Austr. Shepherd	1	Havanese	1
Teckel	2	Dobermann	1	Münsterländer	1
Malteser	2	Whippet	1	Cocker	1
Bernes mountain dog	1	Akita	1	Coton de Tulear	1
Chow chow	1	Appenzeller Mountain Dog	1	Rottweiler	1
Border colie	1	Long hair collie	1	Shar-pei	1
Boston terrier	1	German Quail Dog	1	Goldendoodle	1
Welsh corgi	1	Bichon frisé	1	Leonberger	1
Hungarian Pointer	1	Chihuahua	1		

For the analysis of the complete bones, the first 5 Modes of variations accounted for around 99% of the total variation (Table 3). For the first group of the femur, the first Mode accounted for 93.5% of the total variation with only needing the first 2 Modes to add up to 99.1% of total variation (Table 3). The mean and the standard deviation models for Group 1 and Group 2 are shown in Figures 1–3. For all complete femur groups, size/scale seems to best represent the variation for Mode 1.

**Table 3:** Percentage of variation explained by each mode for each group.

	Complete Femur	Group 1		Group 2		Group 3		Group 4		Group 5		Group 6	
Side	Modes	A	B	A	B	A	B	A	B	A	B	A	B
Left	1	93.5	93.5	91.3	91.3	91.7	91.7	73.5	73.5	93.8	93.8	90.1	90.1
	2	5.62	99.1	6.06	97.3	7.6	99.3	23.6	97.1	5.04	98.9	8.54	98.6
	3	0.34	99.5	1.49	98.8	0.22	99.5	1.08	98.2	0.35	99.2	0.67	99.3
	4	0.17	99.6	0.39	99.2	0.15	99.6	0.82	99	0.28	99.5	0.36	99.6
	5	0.11	99.7	0.25	99.5	0.12	99.8	0.4	99.4	0.13	99.6	0.11	99.7
Right	1	96.4	96.4	74.2	74.2	92.9	92.9	77.1	77.1	89.5	89.5	87.3	87.3
	2	3.01	99.4	19.6	93.8	6.22	99.2	19.6	96.8	6.72	96.2	11.2	98.5
	3	0.2	99.6	3.53	97.3	0.29	99.5	1.53	98.3	2.95	99.2	0.8	99.3
	4	0.14	99.7	1.57	98.9	0.24	99.7	0.65	98.9	0.28	99.5	0.33	99.6
	5	0.07	99.8	0.5	99.4	0.09	99.8	0.35	99.3	0.2	99.7	0.14	99.7

Group composition according to Table 1. For each group: Column A: individual percentage of variation explained, Column B: accumulative percentage of variation explained.

For the analysis of the head and condyles of the femur, the first 5 Modes of variations accounts for around 93.9%–98.0% of the total variation (Table 4). The tables also show the results for the Tibia, Tibia plateau and Patella. For all Groups the first 5 Modes describe more than 93% of the total variance and each impact of following Modes reduces significantly. The results from left and right side are similar. The most visually relevant Modes of the regions of femur, tibia, complete tibia, and patella are shown in Figures 4–6.

**Table 4:** Percentage of variation explained by each mode for each group.

		Femur condyles		Femoral head		Tibia		Tibia plateaus		Patella	
Side	Modes	A	B	A	B	A	B	A	B	A	B
Left	1	80.4	80.4	85.2	85.2	94.7	94.7	78.9	78.9	73.3	73.3
	2	7.46	87.9	3.21	88.4	4.18	98.9	9.13	88	10.8	84.1
	3	3.55	91.5	2.09	90.5	0.61	99.5	4.79	92.8	5.93	90
	4	3.05	94.5	1.92	92.5	0.15	99.7	2.03	94.9	2.4	92.4
	5	0.92	95.4	1.46	93.9	0.1	99.8	1.22	96.1	1.39	93.8
Right	1	67.3	67.3	83.4	83.4	97.6	97.6	77.6	77.6	75.3	75.3
	2	25.6	92.9	5.12	88.5	1.82	99.4	11.3	88.8	9.07	84.4
	3	2.66	95.5	2.35	90.8	0.2	99.6	4.48	93.3	5.06	89.5
	4	1.65	97.2	1.87	92.7	0.09	99.7	1.72	95	2.47	91.9
	5	0.81	98.0	1.43	94.1	0.07	99.8	1.03	96.1	1.82	93.7

For each group: Column A: individual percentage of variation explained, Column B: accumulative percentage of variation explained.

## 4 Discussion

This is the first study to utilize SSM to evaluate and compare the geometric morphology of the femur, tibia, and patella, from dogs without any clinical orthopeadic abnormalities of different sizes and breeds. Eleven groups of analysis were performed using SSM to explore the factors responsible for morphological variability. We found that regardless of size, breed, and bone type, the first Mode showed that the scale factor accounted for most variation (>90% of total variation). Also, it only takes 2–3 Modes to describe more than 99% of total shape variation. In clinical terms, this result suggests that the shape of the bones from a small dog are similar to bones from a larger dog. Another important finding for the femur and tibia was that the second most important source of variation was varus-valgus. Mode 3, 4 and 5 were more difficult to interpret, because the deformation of the shape may depend on multiple factors including shaft diameter and shape, torsion, and epiphysis shape. These results provide a new perspective on how to approach bone morphologic studies on a large scale.

### 4.1 Femur

The second Mode showed a strong varus/valgus deformation of the femur, confirming previous studies that showed strong (up to 4°) variation in normal dogs (9, 40, 41). This result was found in both chondrodystrophic and retriever groups suggesting that varus-valgus is an important anatomical parameter, irrespective of breed. To get further insight on the location of the deformation we realigned the models based on the two extremities and at the mid-diaphysis to observe the curvature of the bone. This study discovered that there is effectively some curvature of the femoral shaft, although it is less than expected. The condyles are also not superimposing, which could also be interpreted as a difference in femoral condyle size.

We found that Modes 3, 4 and 5 were influenced by several factors, which made them more difficult to interpret. It is also important to realize that collectively these 3 Modes accounted only for 0.62% of variation. The best interpretation of the third Mode is a combination of three different components: width of the condyles, femoral head size and craniocaudal thickness of the whole femur. Mode 4 also shows 2 components: procurvation and torsion of the femoral head. Mode 5 could also have thickness and malignment components.

The magnitude of the deformation dependent on Modes 3, 4 and 5 is small compared to the Mode 1 and 2. This is expected because the majority of the variations are from Mode 1. This suggests that when the size factor is eliminated, the normal femur is very similar across different breads. Even though for Groups 2 through Group 6, categorized by weight class and specific breeds, the variability between individual dogs is still dominated by the size/scale factor. This can be identified by the changes in the length of the femur. Group 4 shows the most similar bone length (see Table 1) resulting in less variability in size between Models from large dogs. This reduction of size variability is reflected with the lower percentage represented by Mode 1 compared to other groups. For Group 5, typical specificities of the chondrodystrophic breeds can be seen on the mean model, such as a smaller major trochanter, thicker condyles, or femoral head, and stronger procurvation, which can also be seen on the variant Modes.

To reduce the effect of the femoral length and to isolate the proximal and distal parts of the femur, all the femur models were cut. The only clear interpretation could be made from the first Mode of the femoral condyles SSM, which is the size factor. The rest of the Modes showed a mixture of different combined factors of condyle length, thickness, length of the femoral shaft above the patella grove, and malalignment. Previous studies investigated the femoral condyle size or other condyle variation as a morphological factor that may predispose to CCLD in dogs (42, 43) as well as humans (44, 45). Future studies could use SSM to compare these parameters in dogs with CCLD and normal dogs. For the femoral head SSM, size was the most dominant factor. Interestingly the second Mode represented the size of the femoral head independently of the major trochanter. Modes 3 to 5 represented some differences in alignment as well as a small difference in the anteversion angle of the femoral head. However, the relevance is to be questioned, as the differences are minor. It should be noted that no pathological deformation has been observed such as patellar groove depth differences typically seen in MPL disease, strong varus/valgus of the femoral shaft or deformation of the femoral head typically seen in hip dysplastic cases (18, 20). This indicates a good set of inclusion and exclusion criteria for our data sets.

### 4.2 Tibia

The first 2 Modes from the complete tibia SSM are similar to the femur SSM, representing the size and a combination of varus/valgus variability. While the third Mode showed a combination of different factors, the fourth Mode showed a difference in the craniocaudal thickness of the tibial shaft. The fifth Mode represented a difference in the thickness of the tibia plateau and distal extremity. Interestingly, no Modes show a difference in the position of the tibial tuberosity in relation to the tibial shaft or a torsion like the femur. The lower variability in morphology of the tibia relative to the femur is consistent with previous studies that show that most variation is situated in the femur (46). The Modes from the PCA focused on the tibial plateau follow a similar pattern, with Mode 1 being the size factor. The only other clear factor was observed in Mode 4, procurvation. Other Modes did not show any clear identification of variability.

### 4.3 Patella

The first Mode of the PCA of the patella shows size as the most important factor. Interestingly the second Mode shows an elongation or a more sharply cornered proximal extremity of the patella. Modes three to five most likely represent malalignment.

### 4.4 Interpretation

It should be noted that while some Modes show certain deformations, they can also be interpreted in different ways depending on which Modes are considered. For example, an elongation-type deformation (with a variation on the shaft of the bone only and none on the thickness) can be interpreted as either elongation or as the thickness of the bone, as an elongated bone would be a narrower bone after size adjustment (as the main focus is the proportional variance). Another type of biased interpretation is best shown with the varus/valgus example. If a shaft is seen to be curved while aligned on the condyle, the curvature is not necessarily based on the condyle. It could be situated anywhere on the shaft. Only further analysis with realignment could address this problem.

While the current study does not report measurement of any length or angles, some comparisons can be made with the literature. When reviewing the measurements from different studies on geometrical bone morphology, they show high variability. Additionally, when comparing breeds, the different values overlap between small and large breeds and do not show a clear difference. For example, when looking at the anteversion angle of the femur, some studies found for small breeds angles between 20° and 27°, while on large dogs the values were between 16° and 25° (7, 10, 47–49). While precise angles were not measured in this study, variations on procurvation and torsion which both influence those angles were observed. Stronger procurvation on smaller breeds was observed when comparing different breeds.

Since the number of studies on the tibial angles is limited, the results show more correlation. Smaller breeds have a greater tibia plateau angle (30° vs. 25° in large breeds), Z angle (69.2° to 58.8°), distal tibial axis/proximal tibial axis angle (10.8° to 4.5°), greater relative tibial tuberosity width (0.86° to 0.74°).

We could not see that in our study since we found an overall similarity between breeds. However, the overlapping from those studies is great when looking at the range of the values. Nevertheless, we could show a great variability which could explain the differences seen in those studies. Differences in varus/valgus, procurvation and thickness were observed from the tibia SSM, which all influence those angles (50, 51).

To the best of our knowledge, this study is the first to present SSM for dogs’ hind limbs. This method of measurement considers the whole bone shape rather than single measurements, which is a crucial step toward a better understanding of the anatomy and further research on diseased bones. The different results in measurements in the literature reinforce the need for SSM. One of the tools of SSM is classification, the possibility of classifying if a stifle from a specific case is normal or pathologic bone, without having to rely on individual clinicians’ measurements. With the development of SSMs from bones without any clinical orthopeadic abnormalities, the process can be extended to diseased dogs to observe qualitative and quantitative changes in the Modes for certain diseases such as MPL or HD. This could allow the description of the pathologic changes and the correction needed without the need for manual analysis. Another useful application is using this SSM model to help with the surgery on certain bones by facilitating the finding of landmarks which are needed to perform the operation.

### 4.5 Limitations

This study has several limitations. The most significant factor is the limited sample size. The complete set of femurs (*n* = 61) could be considered a large enough data set, but when divided into groups, the sample sizes for the individual groups are greatly reduced. PCA analysis is a powerful tool that can help represent variations of geometric morphology, but it does not identify a single factor for the significance of each Mode it generates. Therefore, each Mode must be visually analyzed and interpreted. While some of the Modes can be easily identified (Mode 1 for scale), other Modes can be more difficult to interpret due to the significantly smaller magnitude of shape variation presented. Other methods like independent component analysis can be used as an alternative, but they all have their strengths and weaknesses. Also, despite consistent methods of model preparation, errors from segmentation, pre-registration, and alignment cannot be eliminated. Lastly, due to the significant effect of the size variation, other clinically interesting variations of deformation could not be extensively identified. However, this could also be because the current training data sets were from dogs without any clinical orthopeadic abnormalities. And from the chondrodystrophic dog’s half of them were beagle which shows only a small amount of the typical differences seen in those breeds. Perhaps, when data from pathological cases are evaluated, these clinically interesting variations will be observed.

## 5 Conclusion

While SSM is well-known in human medicine, it has only been applied a few times in veterinary orthopedics (52, 53). The goal of this study was to take a further step in this research domain and to establish baseline statistical shape models and values for normal bones. Starting with the stifle (femur, tibia, and patella), SSM was used to evaluate and compare the geometric variation between breed and weight classes for dogs. The results from this study will be used as a reference for further research on investigating geometric morphology for clinically pathological bones and support the advancement of the usage of digital technologies in the veterinary field.

## Data availability statement

The data analyzed in this study is subject to the following licenses/restrictions: all our CTs data are controlled by the University of Zürich. Requests to access these datasets should be directed to bpark@vetclinics.uzh.ch.

## Ethics statement

Ethical approval was not required for the studies involving animals in accordance with the local legislation and institutional requirements because this was a retrospective study. Written informed consent was not obtained from the owners for the participation of their animals in this study because this was a retrospective study.

## Author contributions

JH: Writing – original draft, Writing – review & editing, Conceptualization, Investigation, Methodology. AP: Conceptualization, Project administration, Supervision, Validation, Visualization, Writing – review & editing. JB: Writing – review & editing. T-WL: Writing – review & editing. SK: Conceptualization, Methodology, Supervision, Writing – review & editing. BP: Conceptualization, Data curation, Formal analysis, Investigation, Methodology, Project administration, Software, Supervision, Validation, Visualization, Writing – review & editing.
