# Correction: Statistical shape modeling of the geometric morphology of the canine femur, tibia, and patella

**DOI:** 10.3389/fvets.2026.2000605

**Published:** 2026-09-28

**Authors:** Jeremy Huart, Antonio Pozzi, Jason Bleedorn, Tung-Wu Lu, Sebastian Knell, Brian Park

**Affiliations:** 1 Clinic for Small Animal Surgery, Department for Small Animals, Vetsuisse Faculty University of Zurich, Zürich, Switzerland; 2 Department of Veterinary Clinical Sciences, Colorado State University, Fort Collins, CO, United States; 3 Department of Biomedical Engineering, National Taiwan University, Taipei, Taiwan

**Keywords:** statistical shape model, SSM, morphology, canine, femur, tibia

The funder “University of Zürich Legat Bachofner – Seed Money Experimentelle Chirurgie, Vetsuisse Fakultät Zürich” was erroneously omitted. The correct **Funding** statement reads:

“The author(s) declared that financial support was received for this work and/or its publication. The project received financial support from University of Zürich Lagat Bachofner – Seed Money Experimentelle Chirurgie, Vetsuisse Fakultät Zürich, Switzerland.”

The original version of this article has been updated.

